# The Application of ^29^Si NMR Spectroscopy to the Analysis of Calcium Silicate-Based Cement using Biodentine™ as an Example

**DOI:** 10.3390/jfb10020025

**Published:** 2019-05-30

**Authors:** Qiu Li, Andrew P. Hurt, Nichola J. Coleman

**Affiliations:** 1 State Key Lab of Silicate Materials for Architectures, Wuhan University of Technology, Wuhan 430070, China; qiu-li@whut.edu.cn; 2 Faculty of Engineering and Science, University of Greenwich, Chatham Maritime, Kent ME4 4TB, UK; a.hurt@gre.ac.uk

**Keywords:** Biodentine, calcium silicate cement, endodontic bioceramic, cement hydration, magic angle spinning nuclear magnetic resonance spectroscopy, X-ray diffraction, Fourier transform infrared spectroscopy, calorimetry, nitrogen gas sorption analysis

## Abstract

Biodentine is one of the most successful and widely studied among the second generation of calcium silicate-based endodontic cements. Despite its popularity, the setting reactions of this cement system are not currently well understood. In particular, very little is known about the formation and structure of the major calcium silicate hydrate (C-S-H) gel phase, as it is difficult to obtain information on this poorly crystalline material by the traditional techniques of powder X-ray diffraction analysis (XRD) and Fourier transform infrared spectroscopy (FTIR). In this study, the hydration reactions of Biodentine are monitored by XRD, FTIR, isothermal conduction calorimetry and, for the first time, ^29^Si magic angle spinning nuclear magnetic resonance spectroscopy (^29^Si MAS NMR) is used to investigate the structures of the anhydrous calcium silicate phases and the early C-S-H gel product. XRD analysis indicated that the anhydrous powder comprises 73.8 wt% triclinic tricalcium silicate, 4.45 wt% monoclinic β-dicalcium silicate, 16.6 wt% calcite and 5.15 wt% zirconium oxide. Calorimetry confirmed that the induction period for hydration is short, and that the setting reactions are rapid with a maximum heat evolution of 28.4 mW g^−1^ at 42 min. A progressive shift in the FTIR peak maximum from 905 to 995 cm^−1^ for the O-Si-O stretching vibrations accompanies the formation of the C-S-H gel during 1 week. The extent of hydration was determined by ^29^Si MAS NMR to be 87.0%, 88.8% and 93.7% at 6 h, 1 day and 1 week, respectively, which is significantly higher than that of MTA. The mean silicate chain length (MCL) of the C-S-H gel was also estimated by this technique to be 3.7 at 6 h and 1 day, and to have increased to 4.1 after 1 week. The rapid hydration kinetics of Biodentine, arising from the predominance of the tricalcium silicate phase, small particle size, and ‘filler effect’ of calcite and zirconium oxide, is a favorable characteristic of an endodontic cement, and the high values of MCL are thought to promote the durability of the cement matrix.

## 1. Introduction

Since the introduction of mineral trioxide aggregate (MTA, Dentsply Tulsa Dental, Tulsa, OK, USA) 25 years ago, an expanding range of commercial calcium silicate-based endodontic cements has been brought to market [1,2,3,4]. The original MTA formulation comprised ordinary Portland cement (OPC), to which 20 wt% bismuth oxide (Bi_2_O_3_) was added to enhance radiopacity. OPC is a mixture of impure phases (tricalcium silicate, Ca_3_SiO_5_; dicalcium silicate β-Ca_2_SiO_4_; tricalcium aluminate, Ca_3_Al_2_O_6_; tetracalcium aluminoferrite Ca_2_(Al/Fe)_2_O_5_; and gypsum CaSO_4_·2H_2_O) that undergo complex setting reactions on addition of water [5]. The long setting times (of greater than 2 h) and poor handling characteristics of MTA, in addition to concerns arising from the toxicity of the aluminium-bearing phases, stimulated the development of a second generation of cements based upon mixtures of pure tricalcium and dicalcium silicates (viz. C_3_S and C_2_S, respectively) [1]. Alternative radiopacifying agents to bismuth oxide have also been sought owing to its potential cytotoxicity and deleterious impact on setting times, porosity, mechanical strength and durability of the cement matrix [6,7,8]. 

Biodentine (Septodont, Saint-Maur-des-Fossés, France) is arguably the most popular among the new generation of calcium silicate cements and, accordingly, its properties and clinical applications have been widely reported and reviewed in the scientific literature [1,2,3,4,9,10,11]. It is used, inter alia, in direct and indirect pulp capping, apexifications, retrograde restorations, perforations, resorptions and various other endodontic procedures to repair dentine and cementum [1,2,3,4,9,10,11,12]. The biocompatibility, bioactivity, cytotoxicity and antimicrobial properties of Biodentine are generally reported to be comparable with those of MTA; however, Biodentine is noted for its markedly reduced setting times, improved handling properties and reduced tooth discoloration [1,9,13,14].

The manufacturer’s scientific file states that Biodentine powder comprises a mixture of tricalcium and dicalcium silicates (i.e., the cementing phases), calcium carbonate and oxide (fillers), iron oxide (pigment) and zirconium oxide (radiopacifier) [12]. The accompanying aqueous solution contains dissolved calcium chloride (accelerator) and a hydrosoluble polymer water-reducing agent (i.e., a polycarboxylate superplasticizer). The short setting time of 12 min was achieved by increasing the proportion of the fast-setting C_3_S phase (relative to that of MTA), reducing the particle size and water content and introducing calcium chloride to accelerate the reactions. The replacement of bismuth oxide radiopacifier with zirconium oxide may also contribute to a reduction in setting time, as this material has been shown to accelerate the initial hydration reactions of OPC [15]. The smaller particle size, reduced water content and incorporation of a superplasticizer also improve the handling of Biodentine. 

Strategies to enhance the mechanical, antimicrobial, radiopaque and bioactive characteristics of Biodentine via the incorporation of various admixtures to the proprietary material have been reported recently [16,17,18,19,20,21,22]. These admixtures include alkali-resistant glass fibers [16], titanium tetrafluoride [17], calcium tungstate and additional zirconium oxide [18], casein phosphopeptide-amorphous calcium phosphate (CPP-ACP) [19,20] and various bioactive glasses [21,22]. In most of these studies, owing to the complex nature of cement chemistry, incremental improvements in a particular characteristic are accompanied by the deterioration of other important and clinically relevant properties. Successful modifications to existing calcium silicate-based cements and the development of the next generation of these materials require a comprehensive understanding of the hydration and setting reactions of the calcium silicate phases to ensure the safe, effective and predictable clinical performance of these materials. 

**Figure 1:**
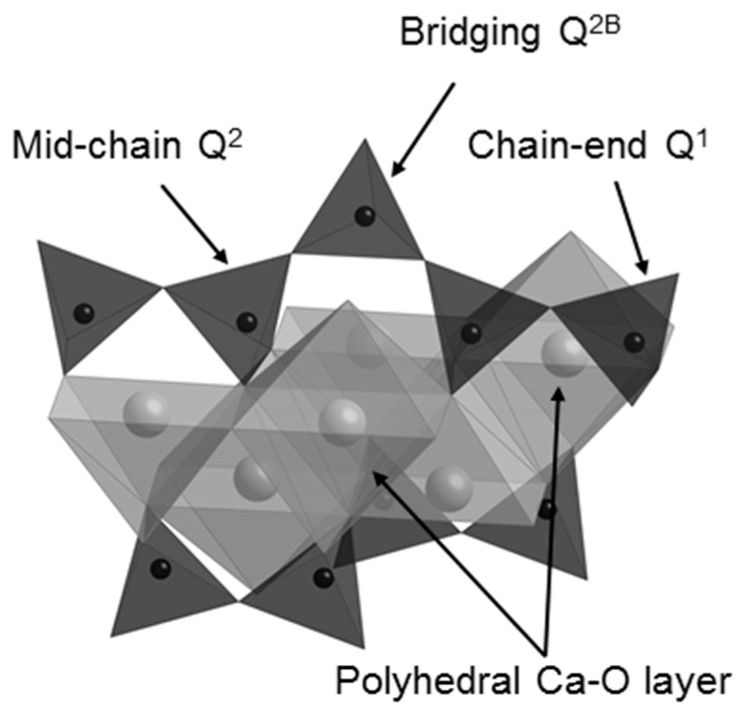
The structure of the calcium silicate layer of the C-S-H gel phase (water molecules and hydroxyl groups are not shown).

On mixing with water, the C_3_S and C_2_S phases of Biodentine undergo exothermic hydration reactions to form hexagonal crystals of calcium hydroxide and an adhesive calcium silicate hydrate (C-S-H) gel network that results in the setting of the mixture [5,15,23,24]. C-S-H is a poorly crystalline, layered, nanoporous phase of nonstoichiometric composition. Capillary water resides in the nanopores of the C-S-H gel and free residual mix water resides in the meso- and macropores within the hydrated cement matrix. C-S-H ideally comprises double layers of calcium oxide polyhedra linked on both sides to silicate chains, as shown in Figure 1. During hydration, anhydrous unpolymerized isolated (Q^0^) silicate tetrahedra in C_3_S and C_2_S are hydroxylated (Q^0^(H)) by the mix-water, then dissolve and subsequently condense together to form dimers (Q^1^) [5,15]. Further condensation leads to the formation of short silicate chains comprising mid-chain (Q^2^) species facing into the calcium oxide layer, bridging species (Q^2B^) that link the Q^2^ species, and chain-end (Q^1^) groups. 

The overall hydration reactions of C_3_S and C_2_S at ambient temperature are given in Equations (1) and (2) [24]:

Ca_3_SiO_5_ + 5.2H_2_O→1.3Ca(OH)_2_ + (CaO)_1.7_SiO_2_(H_2_O)_4_ (1)

Ca_2_SiO_4_ + 4.3H_2_O→0.3Ca(OH)_2_ + (CaO)_1.7_SiO_2_(H_2_O)_4_ (2)

The hydration reactions of C_3_S and C_2_S proceed via several stages of dissolution and subsequent precipitation of calcium hydroxide and C-S-H gel. Tricalcium silicate, the major cementing phase in Biodentine, reacts more rapidly than dicalcium silicate and consequently controls the setting behavior, heat evolution, strength-development and durability of the resulting cement. The other mineral constituents of Biodentine do not formally participate in the hydration reactions, although calcium carbonate and zirconium oxide are known to present nucleation sites for the precipitation and growth of the early C-S-H gel products, which accelerate the initial setting reactions (aka the ‘filler effect’) [15,25]. 

The traditional technique for the investigation of the early kinetics of the hydration of C_3_S-based cements is isothermal conduction calorimetry, which monitors heat evolution as a function of time [23,24]. This technique is commonly used to evaluate the accelerating or retarding impact of additives, and the effects of particle size, water content and temperature on cement systems [5,23,24,26,27]. 

The most widely used instrumental techniques for the analysis of the composition and hydration chemistry of calcium silicate dental cements are: scanning electron microscopy (SEM) to investigate microstructure [21,28,29,30,31,32,33]; energy dispersive X-ray analysis (EDX) for elemental composition and mapping [21,28,29,30,31,32,33,34]; Fourier transform infrared (FTIR) and Raman spectroscopies to characterize constituent functional groups [5,8,21,22,30,31,33,34,35]; and powder X-ray diffraction analysis (XRD) for the determination of the crystalline phases [5,21,22,28,30,33,36]. Of these techniques, FTIR spectroscopy and XRD analysis are the most commonly used to determine the chemical structures of the components of anhydrous and hydrated cements. 

FTIR spectroscopy is a rapid technique that can be used to detect the presence of anhydrous C_3_S and C_2_S and to monitor the development of the C-S-H gel via the shift to higher wavenumbers of the O-Si-O stretching vibrations as polymerization proceeds [37]. Some semi-quantitative analysis can be carried out, but this is complicated by the bands arising from the presence of capillary and free water and calcium carbonate. Powder XRD analysis in combination with Rietveld refinement is routinely used in the cement industry for the quantitative analysis of clinkers and anhydrous cements [38,39]; however, the poorly crystalline nature of the C-S-H gel phase prohibits its comprehensive structural analysis by this technique. 

Surprisingly, to date, very few studies of proprietary and experimental calcium silicate-based endodontic cements have utilized the powerful analytical technique of ^29^Si magic angle spinning nuclear magnetic spectroscopy (MAS NMR) to investigate the hydration and setting reactions of C_3_S and C_2_S [5,15,40,41]. In contrast, ^29^Si MAS NMR is a well-established and widely applied tool in general cement science and technology [23,42,43,44,45,46,47]. This technique is sensitive to the local chemical environment of the silicate tetrahedra within the anhydrous phases and C-S-H gel. Unlike XRD analysis, it is effective for the structural determination of both crystalline and amorphous materials as it does not rely on long-range order. Using simple spectral deconvolution, ^29^Si MAS NMR can be used to discriminate among the various Q^n^ species to enable the calculation of degree of hydration and average silicate chain length of the C-S-H gel [23,42,43,44,45,46,47]. Accordingly, this technique has the potential to provide valuable information on the effects of cement composition, particle size, additives and contaminants on the structure of calcium silicate-based endodontic cements that cannot be obtained by other instrumental methods. 

In the current literature, reports of the application of ^29^Si MAS NMR to the composition and setting of glass ionomer cements are numerous [48,49,50,51,52,53,54,55], yet those of calcium silicate-based dental cements are limited to one study of ProRoot MTA [5] and a few others on the impact of radiopacifying zirconium oxide [15,40], iodoform [41] and bismuth oxide [56] on experimental OPC-based materials. 

Since, to date, there are no reports on the analysis of Biodentine by ^29^Si MAS NMR, the principal objective of this present study was to use this technique to investigate the structures of the anhydrous C_3_S and C_2_S phases and the early C-S-H gel product after 6 h, 1 day and 1 week. The as-received Biodentine powder and hydrated samples were characterized by EDX, nitrogen gas sorption analysis, XRD, FTIR and ^29^Si MAS NMR, and the reaction kinetics were also monitored using isothermal conduction calorimetry to provide a comprehensive account of the hydration chemistry of this popular endodontic restorative. 

## 2. Results

### 2.1. SEM/EDX and Gas Sorption Analysis

**Figure 2:**
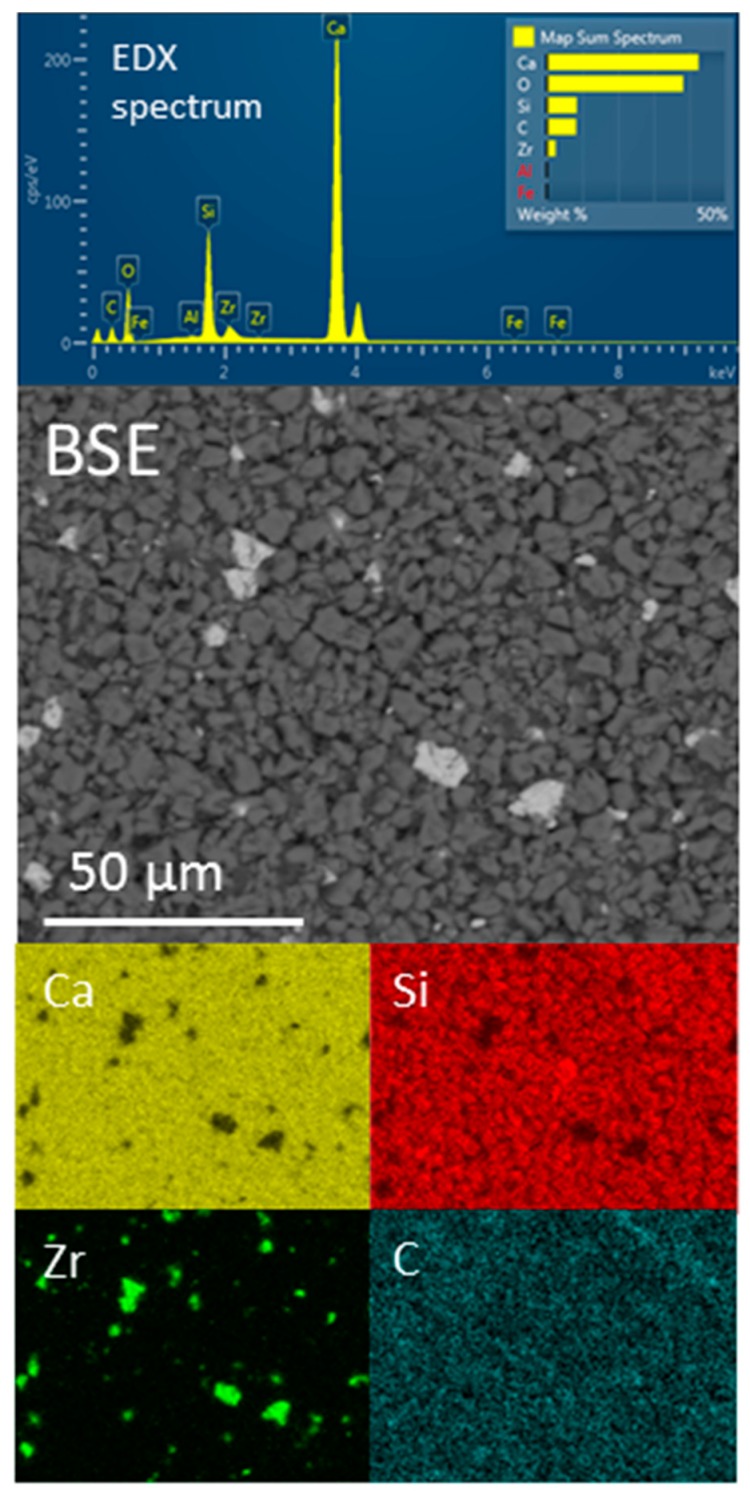
EDX spectrum, backscattered electron (BSE) image (×100) and EDX maps of calcium, silicon, zirconium and carbon for anhydrous Biodentine powder.

A backscattered electron (BSE) image, the EDX spectrum and elemental maps of calcium, silicon zirconium and carbon of a compact of anhydrous Biodentine powder are shown in Figure 2. EDX analysis estimates the average composition of the compact to be 42.6 wt% Ca, 8.4 wt% Si, 8.2 wt% C, 2.5 wt% Zr and 38.3 wt% O, with trace quantities of Fe and Al below 0.1 wt%, which is in agreement with data reported previously [32,33]. The BSE image shows that this material is a finely divided powder comprising irregularly shaped particles with a broad size distribution (Figure 2). The majority of the particles are in the approximate size range 1–6 μm, with a significant fraction of sub-micron-sized powder and some larger particles. Comparison of the BSE image and elemental maps for Ca, Si and Zr show discrete 2–10 μm radiopacifying zirconium oxide particles distributed among the calcium silicate cement phases, and the elemental map for C indicates that calcium carbonate is present as a finely divided sub-micron sized powder. SEM and EDX data of hydrated samples are not presented in this study, as the application of these techniques to the microstructural analysis of hydrated Biodentine is extensively documented in the current literature [21,28,29,30,31,32,33]. 

The specific surface area of anhydrous Biodentine powder was determined by nitrogen gas sorption and BET analysis [57]. It was found to be 3.35 ± 0.02 m^2^ g^−1^, which is intermediate between the values of 2.8 m^2^ g^−1^ and 4.0 m^2^ g^−1^ reported in other studies [58,59]. The specific surface area subsequently increased to 30.4 ± 0.1 m^2^ g^−1^, 30.5 ± 1.4 m^2^ g^−1^ and 30.5 ± 0.1 m^2^ g^−1^ following hydration at 37 °C for 6 h, 1 day and 1 week, respectively. The 10-fold increase after 6 h is attributed to the rapid development of the C-S-H gel phase and the formation of the porous cement matrix. 

### 2.2. XRD Analysis 

**Figure 3:**
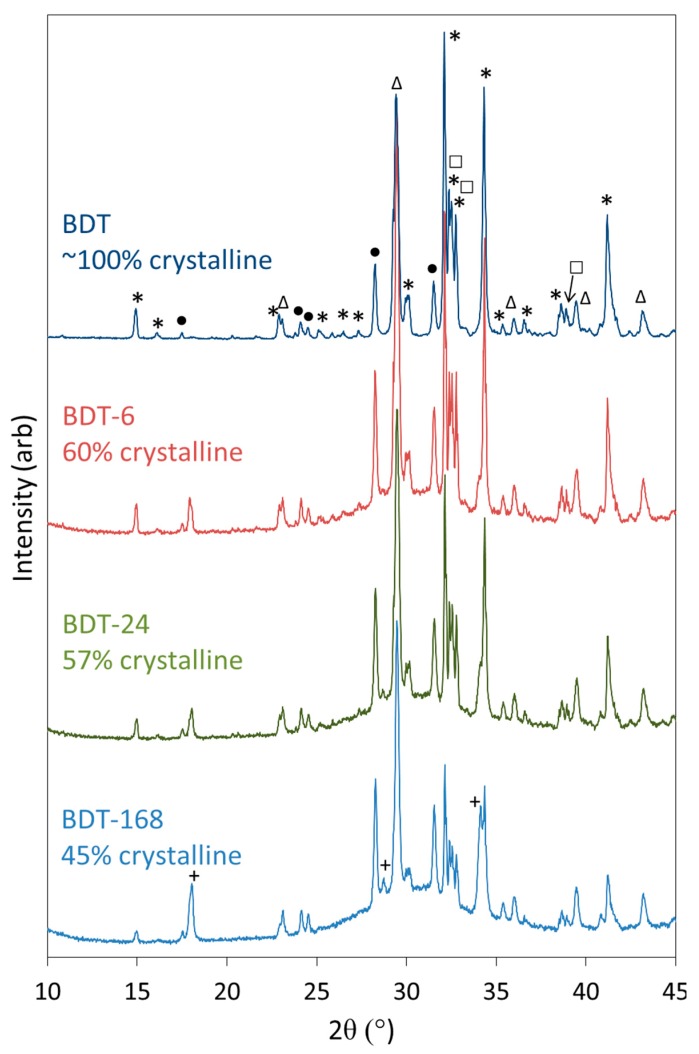
XRD patterns of anhydrous Biodentine (BDT) and Biodentine hydrated for 6, 24 and 168 h (BDT-6, BDT-24 and BDT-168, respectively) (*—C_3_S; □—C_2_S; •—ZrO_2_; ∆—CaCO_3_ and +—Ca(OH)_2_).

Powder XRD data were collected for anhydrous Biodentine and for the hydrated specimens to confirm the initial composition of the material and determine the crystalline phases arising from the hydration reactions (Figure 3). The structures and proportions of the phases present in the anhydrous powder were also determined using Rietveld refinement analysis, for which a weighted profile R-factor (R_wp_) of 10.8 and goodness of fit (GOF) parameter of 2.7 were obtained, and demonstrate that the fitting was satisfactory [60]. The anhydrous powder was found to comprise 73.8 wt% triclinic C_3_S, 4.45 wt% monoclinic β-C_2_S, 16.6 wt% calcite and 5.15 wt% zirconium oxide, with a total crystallinity of approximately 100%. Contrary to the manufacturer’s scientific file [12], reflections corresponding to calcium oxide and iron oxide were not detected by this technique and indicate that these components were present below the limit of detection (<1 wt%), if at all. 

Qualitative powder XRD data (Figure 3) show that the reflections for C_3_S and C_2_S diminish as hydration proceeds and these phases react to form C-S-H gel and portlandite. The development of the reflections of portlandite is clearly observed; however, those of the C-S-H gel are not apparent as this phase is highly disordered. The formation of C-S-H gel is evidenced by the rise in the baseline in the 2θ range between 25° and 35° and in the concomitant decrease in crystallinity of the sample as functions of time. 

The reflections of calcite and zirconium dioxide persist in the diffraction patterns throughout the 7-day period, and there is no evidence of the formation of any other carbonate- or zirconium-bearing phases within this timeframe, indicating that these components do not formally participate in the hydration reactions. 

### 2.3. FTIR Spectroscopy

**Figure 4:**
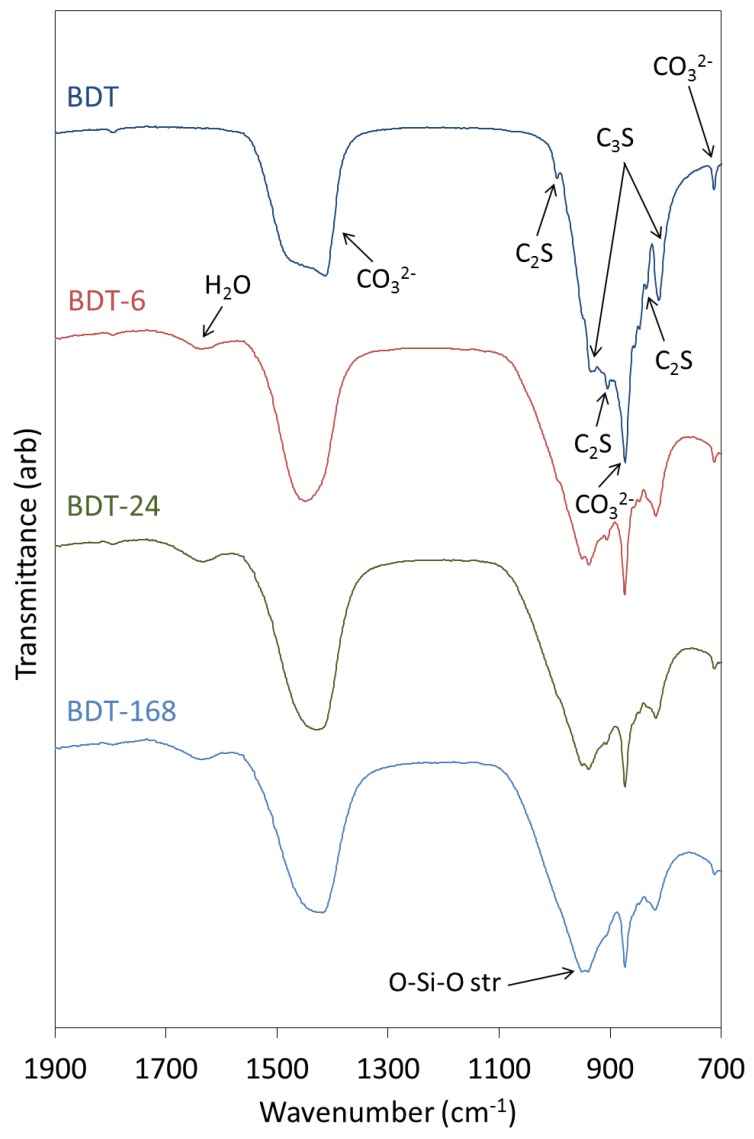
FTIR spectra of anhydrous Biodentine (BDT) and Biodentine hydrated for 6, 24 and 168 h (BDT-6, BDT-24 and BDT-168, respectively).

The FTIR spectra of anhydrous and hydrated Biodentine obtained in this study (Figure 4) are similar to those reported by other researchers [21,22,35]. In the spectrum of the anhydrous powder, C_3_S is denoted by O-Si-O and Si-O-Ca stretching modes at 813 and 936 cm^−1^, respectively [31,37]. Bands at 905 and 995 cm^−1^ are assigned to Si-O-Ca stretching vibrations of C_2_S, and this phase gives rise to O-Si-O stretching modes at 840 cm^−1^ [31,37]. Various vibrations arising from the carbonate group of calcite appear at 714, 875 and 1445 cm^−1^. 

During the hydration of calcium silicate-based cements at ambient temperature, the various O-Si-O stretching bands typically broaden and shift to higher wavenumbers with the increasing polymerization of the silicate chain structure of the C-S-H gel. Accordingly, as the hydration of Biodentine proceeds, the signals arising from the various stretching modes of C_3_S and C_2_S are observed to diminish as these phases are consumed. Throughout the 1-week hydration period, the ongoing condensation of the C-S-H gel phase is denoted by the progressive shift in the signal maximum of the O-Si-O combination stretching band from 905 to 995 cm^−1^. Bending modes of hydrogen-bonded hydroxyl groups and of free and capillary water are also observed at 1640 cm^−1^ in the spectra of all hydrated samples. 

Vibrational modes of the zirconium oxide lattice do not appear in the reported spectral region and, thus, do not complicate the interpretation of the spectra [8,26]. It should also be noted that the organic polycarboxylate superplasticizer in Biodentine is not present in sufficient concentration to be detected by this technique. 

### 2.4. Isothermal Conduction Calorimetry

**Figure 5:**
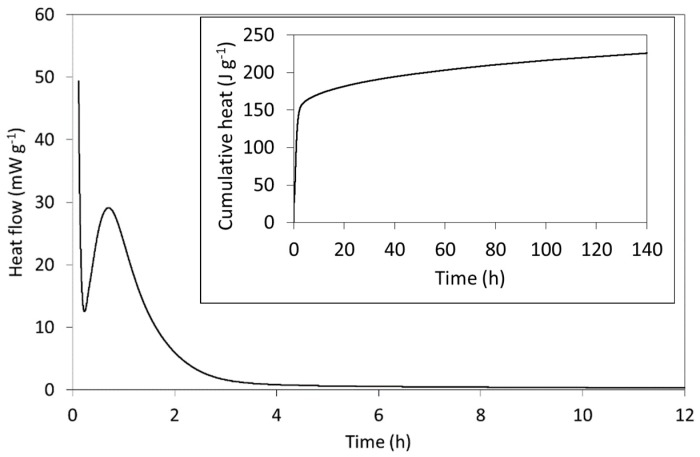
The initial rate of heat evolution and (inset) the cumulative heat released from Biodentine during hydration at 37 °C.

The rate of heat evolution per gram of freshly mixed Biodentine paste (including both powder and solution) and the cumulative heat evolved (inset) are plotted in Figure 5 and compare well with those of other C_3_S-based cements reported in the literature [23,24,61]. The setting reactions of Biodentine are exothermic, and the heat evolved is principally dictated by the hydration of the major tricalcium silicate phase. In addition to its greater abundance, its reaction kinetics are more rapid than those of the minor C_2_S phase and its enthalpy of hydration (−121 kJ mol^−1^) is considerably higher than that of C_2_S (−43 kJ mol^−1^) [24]. 

As indicated by the rate of heat evolution (Figure 5), Biodentine hydration is characterized by a sequence of stages that typify those of C_3_S-based cements [24]. The first involves the highly exothermic wetting and dissolution of the surface calcium, oxide and silicate ions within a few minutes of mixing. The initial formation of hydration products then precedes a dormant (induction) phase during which the dissolution and precipitation reactions slow and heat evolution is reduced. The dormant phase can vary between a few minutes and several hours, depending on the particle size, temperature, water content and the nature of any additives. In this case, the dormant phase occurs ~17 min after mixing and is short, owing to the small particle size, presence of calcium chloride accelerator and modestly elevated temperature of 37 °C (i.e., body temperature rather than 20 or 25 °C, which are more commonly reported for calorimetric measurements of cements). The subsequent acceleration period is then governed by the growth of the C-S-H gel product phase with a maximum rate of heat evolution of 28.4 mW g^−1^, which occurs 42 min after mixing. The deceleration stage that follows marks a decline in hydration rate as the reactions become diffusion-controlled. The specific mechanisms of this familiar sequence of hydration stages of C_3_S-based cements remain disputed despite being the subject of intensive scientific enquiry for over a century [23,24,61]. 

### 2.5. ^29^Si MAS NMR Spectroscopy

^29^Si MAS NMR spectroscopy is used to determine the local environment and extent of polymerization of Q^n^ silicate species in a wide range of amorphous and crystalline materials [62]. In ^29^Si MAS NMR spectra, the intensity of the various Q^n^ signals are plotted against a ‘chemical shift’ scale, which is referenced to a standard (usually tetramethylsilane, TMS). Spectra can be obtained such that the intensity of the various signals is proportional to the relative abundance of the Q^n^ species, although the signals are often overlapping and require deconvolution. 

Isolated Q^0^ silicate units, such as those of C_3_S and C_2_S, have a chemical shift range of −65 to −75 ppm, and Q^1^ species give rise to signals in the range −78 to −82.5 ppm. Signals of Q^2^ silicate tetrahedra occur between −84 and −87.5 ppm, with bridging Q^2B^ species appearing approximately 5 ppm higher (i.e., less negative) than the mid-chain Q^2^ units facing into the calcium oxide layer of the C-S-H gel (Figure 1) [45,46]. 

**Figure 6:**
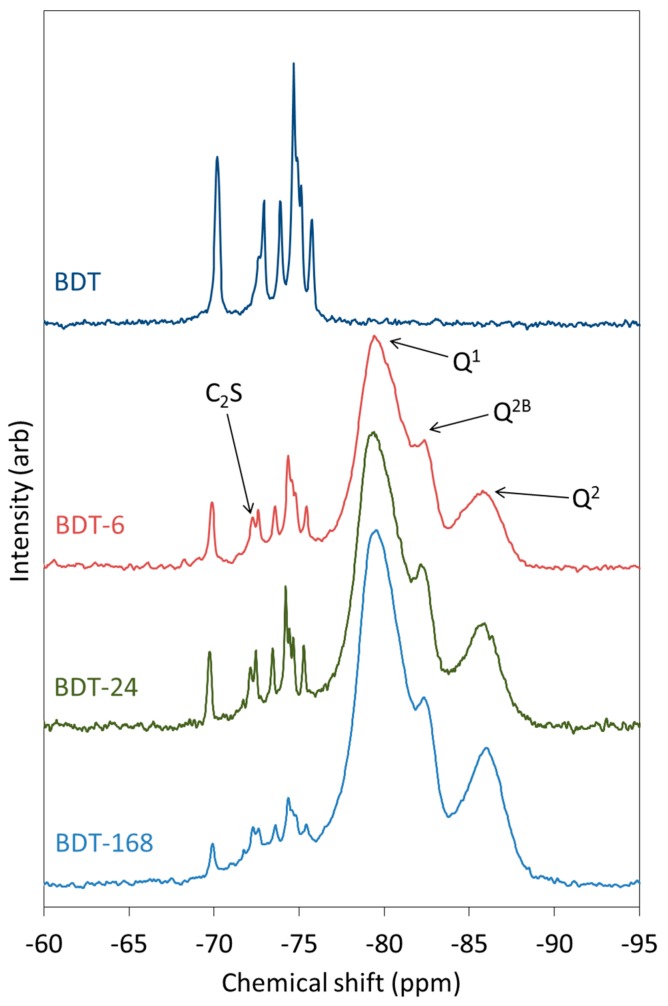
^29^Si MAS NMR spectra of anhydrous and hydrating Biodentine.

The ^29^Si MAS NMR spectra of anhydrous and hydrating Biodentine are shown in Figure 6. The spectrum of pure triclinic C_3_S is known to display up to nine resolved peaks between −66.5 and −74.5 ppm that represent the crystallographically distinct Q^0^ silicate environments in this structure, whereas β-C_2_S presents a single resonance at approximately −71 ppm [63,64]. In the spectrum of anhydrous Biodentine, the C_3_S phase gives rise to signals at −69.7, −71.7 (unresolved shoulder), −72.6, −73.6, −74.3, −74.4 (shoulder), −74.6 and −75.3 ppm. In this case, the signal at −69.7 ppm is unresolved, although it is reported to comprise two overlapping peaks at −68.9 and −69.0 ppm [63]. The single resonance of C_2_S is observed at −72 ppm [63]. These data indicate that both the C_3_S and C_2_S phases of Biodentine are pure and crystalline. 

As hydration proceeds, peaks corresponding to Q^1^, Q^2^ and Q^2B^ silicate species of the C-S-H gel appear and intensify at the expense of the Q^0^ signals. After just 6 h, the peaks arising from the C-S-H gel account for a considerable proportion of the intensity of the spectrum and demonstrate the very rapid rate of hydration of this material. The rate of disappearance of the peaks corresponding to C_3_S is more rapid than that of C_2_S. 

### 2.6. ^1^H-^29^Si CP MAS NMR Spectroscopy

^1^H-^29^Si cross polarization (CP) MAS NMR spectroscopy is used to discriminate between anhydrous and hydrated silicate species in cement systems, as anhydrous species do not appear in the spectrum [62]. The chemical shift ranges for ^1^H-^29^Si CP MAS NMR spectra are concurrent with those given in the previous section for single-pulse ^29^Si MAS NMR spectroscopy, although the intensities of the resonances obtained using this technique are not proportional to the relative abundance of the various Q^n^ species within the sample. 

**Figure 7:**
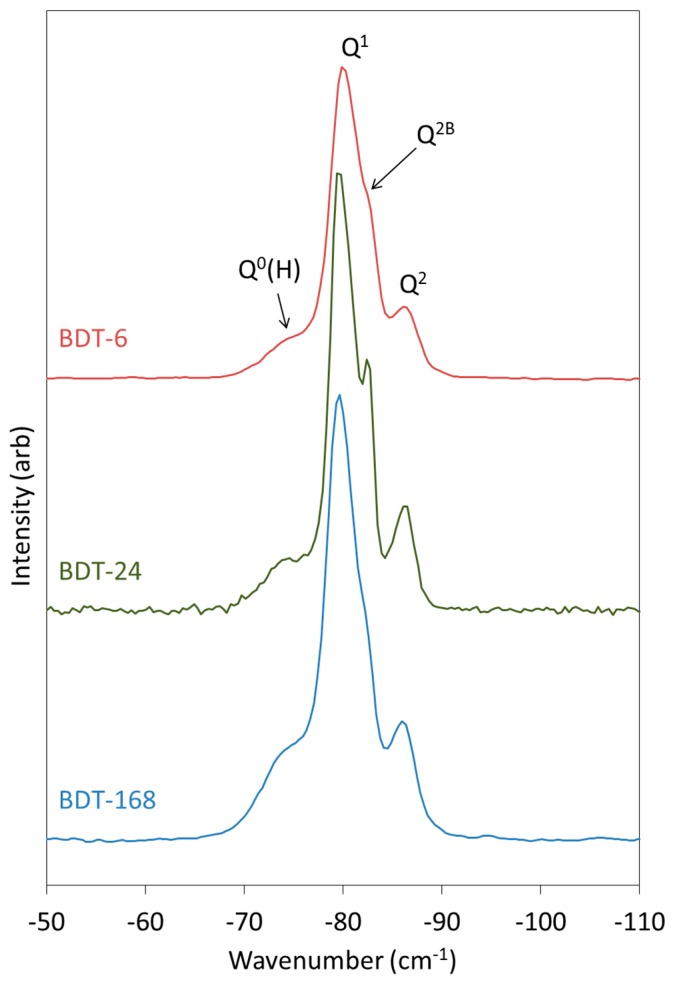
^1^H-^29^Si CP MAS NMR spectra of hydrating Biodentine.

The ^1^H-^29^Si CP MAS NMR spectra of the hydrating Biodentine samples are presented in Figure 7. These spectra were obtained to determine the chemical shifts of the early Q^0^(H) and Q^1^ hydration products, as these signals are partially obscured by those of C_3_S and C_2_S in the corresponding single-pulse ^29^Si MAS NMR spectra (Figure 6). After 6 h of hydration, Q^0^(H), Q^1^ and Q^2^ resonances are detected at approximately −73, −80 and −85.5 ppm, with a shoulder on the Q^2^ signal at −82.5 arising from bridging Q^2B^ silicate tetrahedra [65]. 

### 2.7. Deconvolution and Quantitative Analsis of ^29^Si MAS NMR Spectra

**Figure 8:**
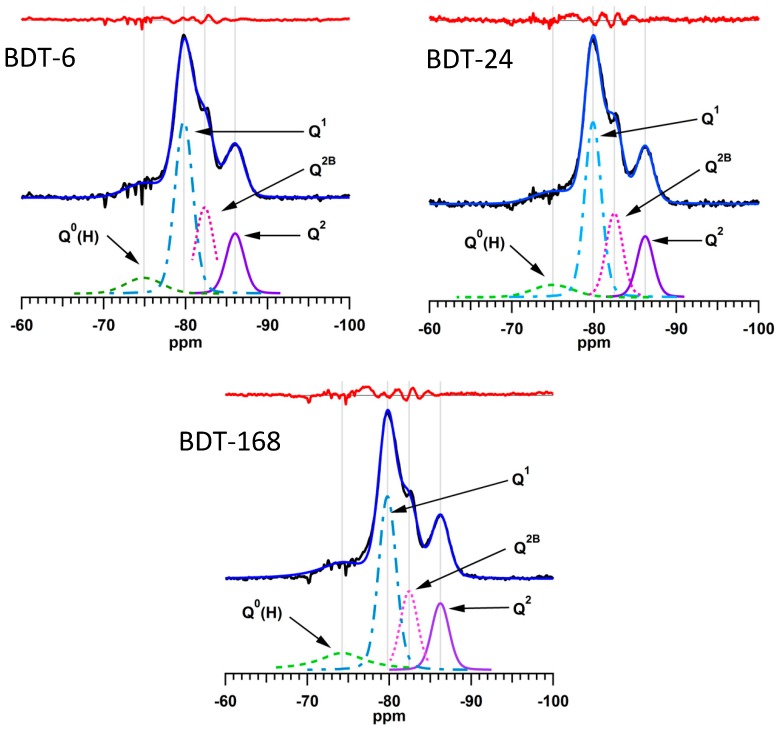
Subtracted, fitted and deconvoluted ^29^Si MAS NMR spectra of hydrating Biodentine.

The ^29^Si MAS NMR spectra of the hydrating Biodentine samples were analyzed by a method reported by Love et al. [45] in which the signals from the unreacted C_3_S and C_2_S that obscure the resonances of the early hydration products are subtracted from the spectrum prior to deconvolution. The subtracted, deconvoluted and calculated ^29^Si MAS NMR spectra of the hydrating Biodentine samples are shown in Figure 8. The residues (i.e., the differences between the subtracted and calculated spectra) are plotted in red above the corresponding spectra. The low intensities of the residues confirm the validity of this method for the deconvolution and quantitative analysis of these spectra. 

Experimental and computational studies indicate that the number of condensed silicate tetrahedra, *m*, present in the individual silicate chain sequences of C-S-H gel obeys the empirical formula *m* = 3*n* − 1, where *n* is a positive integer [64,66]. This suggests that, during hydration, silicate dimers (i.e., *n* = 1) are initially precipitated, then merge together with other dimers and monomers to form stable pentamers (*n* = 2). The subsequent formation of octamers (*n* = 3) has also been reported [66]. 

The relative abundance of Q^n^ species, degree of hydration and mean silicate chain length of the C-S-H gel of the hydrating Biodentine samples are listed in Table 1. These data confirm the rapid hydration of this material (i.e., 87% within 6 h) in comparison to that of MTA, which is 10% hydrated after 6 h and only achieves ~60% hydration within 1 week [5]. 

The mean silicate chain lengths of the C-S-H gel phase of Biodentine at all observed times (Table 1) are higher than the value of 2.7 reported for a pure C_3_S cement of similar particle size that was hydrated for 16 days at 20 °C [23]. Mean silicate chain length is highly significant to calcium silicate-based endodontic cements, as longer silicate chains promote the durability of the cement matrix [66]. 

## 3. Discussion

In order to modify and develop the next generation of calcium silicate-based dental cements, it is necessary to have a comprehensive understanding of their hydration chemistry. Knowledge of the impact of additives, irrigants, acids, medicaments, body fluids and other contaminants and contacting dental restoratives on the hydration and setting reactions is also essential to the development and effective clinical application of these materials [67]. 

To date, the microstructure and composition of Biodentine and the evolution of the C-S-H gel and calcium hydroxide product phases have been widely investigated by SEM and EDX and are well documented in the literature [21,28,29,30,31,32,33]. Accordingly, the SEM and EDX data collected in this study for the anhydrous powder component of Biodentine confirm those reported by other researchers [32,33]. Conversely, there are fewer reports on the semi-quantitative analysis of Biodentine by XRD, and these are not in agreement. For example, Camilleri et al. [58] report that the anhydrous powder comprises 80.1 wt% monoclinic C_3_S, 14.9 wt% calcium carbonate and 5.0 wt% zirconium oxide, whereas Grazziotin-Soares et al. [36] state that it is composed of monoclinic and triclinic C_3_S at 23.8 and 56.6 wt%, respectively, 14.9 wt% calcium carbonate and 4.7 wt% zirconium oxide. Grazziotin-Soares et al. [36] also report that C_2_S was not found in the anhydrous material, but that it was present in the sample that had been hydrated at 37 °C for 1 week. It is not possible for C_2_S to form under these conditions, and so this finding casts doubt over the validity of the refinement method used in this study.

The present research indicates that anhydrous Biodentine powder comprises triclinic C_3_S, monoclinic β-C_2_S, calcite and zirconium oxide, and the presence of the β-C_2_S phase is also confirmed by its characteristic vibrations at 995, 905 and 840 cm^−1^ in the FTIR spectrum and diagnostic resonance at −72 ppm in the ^29^Si MAS NMR spectrum [31,37,63]. It is unclear whether the reported compositional differences of the anhydrous powder of Biodentine arise from inter-batch variations or difficulties in obtaining accurate information from XRD analysis arising from the extensive overlap of the C_3_S and C_2_S reflections. This notwithstanding, the present study has demonstrated that FTIR and ^29^Si MAS NMR are useful supplementary techniques for detecting the presence of, and discriminating between, the C_3_S and C_2_S phases. 

As mentioned, owing to the highly disordered structure of C-S-H gel, XRD analysis is not able to yield structural information on this important product phase, and FTIR spectroscopy is also of limited use [37,43]. Using these techniques, the development of the C-S-H phase is observed by XRD as a progressive decrease in the degree of crystallinity of the sample and by an increase in the peak maximum of the O-Si-O stretching vibrations in the FTIR spectrum. The present study has shown that ^29^Si MAS NMR spectroscopy is an important technique for the analysis of this phase, as it can reveal quantitative information on the different Q^n^ environments, degree of hydration and mean silicate chain length. In addition, ^1^H-^29^Si CP MAS NMR spectroscopy can be used to distinguish between anhydrous and hydrated silicate species. 

A previous ^29^Si MAS NMR study on the hydration of ProRoot MTA reports that 40% reacts within the first 24 h, and that the hydration reactions are ~60% complete within 1 week [5]. In comparison, the extent of hydration of Biodentine was determined to be 87.0%, 88.8% and 93.7% at 6 h, 1 day and 1 week, respectively, which is significantly higher than that of MTA. The superior setting kinetics of Biodentine have also been observed by isothermal conduction calorimetry. The hydration of MTA involves a 4-h induction period shortly after mixing, and a maximum heat evolution at the peak of the acceleration stage at 16 h. In contrast, the induction period of Biodentine ends after ~20 min, and the peak maximum of the acceleration stage occurs 42 min after mixing. 

There are no current reports on the MCL of the C-S-H gel of commercial calcium silicate cements. However, one study found that the incorporation of 20 wt% iodoform radiopacifier in an experimental white Portland cement had little impact on the rate of hydration, although it did cause a reduction in MCL from 4.11 to 3.47 units at 1 week [41]. The MCL of 4.13 observed for the week-old C-S-H gel of Biodentine indicates a tendency towards the formation of silicate pentamers rather than dimers within the structure that promote the durability and reduce the solubility of the cement matrix [66].

This study has demonstrated that, when used in combination, XRD analysis, FTIR and ^29^Si MAS NMR spectroscopies and isothermal conduction calorimetry can provide comprehensive insight into the hydration chemistry of calcium silicate-based endodontic cements. The information provided by these techniques will facilitate further modifications and developments of these materials by enabling essential understanding of the complex relationships between composition, setting reactions and subsequent structural evolution and clinical performance. ^29^Si MAS NMR spectroscopy is of particular relevance for the elucidation of the chemistry of the major C-S-H gel product. Accordingly, this technique has the potential to provide valuable information on the effects of cement composition, particle size, additives and contaminants on the structure of calcium silicate-based endodontic cements that cannot be obtained by other instrumental methods. 

## 4. Materials and Methods

### 4.1. Preparation of Biodentine Samples

Biodentine (Septodont, Saint-Maur-des-Fossés, France) samples were prepared according to the manufacturer’s instructions. The resulting pastes were sealed in polypropylene containers and cured at 37 °C for 6, 24 or 168 h. Each sample type (viz. BDT-6, BDT-24 and BDT-168) was prepared in duplicate. Prior to analysis by powder XRD, FTIR and ^29^Si MAS NMR, the hydration reactions were stopped by solvent exchange with propan-2-ol. This was achieved by immersion of 2-mm fragments of the pastes in four consecutive 50 cm^3^ washings of propan-2-ol in a sonic bath for 30 min. The samples were then dried to constant mass in a vacuum desiccator at room temperature. 

### 4.2. SEM/EDX Analysis

As-received, anhydrous Biodentine powder was pressed flat using a die press, and the resulting compact was attached to a carbon tab. A backscattered electron image at a magnification of ×100 was obtained from the uncoated sample using a JEOL JSM-5410 LV scanning electron microscope (JEOL, Tokyo, Japan) with an Oxford Instruments X-MaxN EDX detector (Oxford Instruments, Abingdon, UK) in low vacuum mode using an accelerating voltage of 20 kV. An EDX spectrum and elemental maps for calcium, silicon, zirconium and carbon were collected over the entire field of view (1.29 × 0.94 mm) using a working distance of 20 mm. 

### 4.3. Nitrogen Gas Sorption Analysis

The specific surface area of the anhydrous Biodentine powder was obtained by nitrogen gas sorption analysis via the BET method [57]. Prior to nitrogen sorption, the sample was heated at 40 °C under flowing helium gas to remove physically adsorbed material from its surface. The analysis was performed on a Micromeritics Gemini V gas sorption analyzer (Micromeritics Instrument Corporation, Norcross, GA, USA). Nitrogen gas of 99.999% purity was used to collect seven adsorption points in the relative pressure range 0.05 < P/P_0_ < 0.30 (where P_0_ is the saturated vapor pressure) at 77.4 K that were used to calculate the BET surface area, taking the cross sectional area of adsorbed nitrogen molecules to be 0.162 nm^2^. 

### 4.4. XRD Analysis

Powder XRD analysis was performed on the anhydrous Biodentine powder (labelled ‘BDT’) and on all hydrated specimens using a Bruker D8 diffractometer (Bruker AXS, Karlsruhe, Germany) with Cu Kα = 1.5406 Å, a step size of 0.019° in the 2θ range from 10° to 45° and a measuring time of 1 s per step. X-ray diffraction data were compared with Powder Diffraction Files (PDF) using DIFFRAC.EVA software (supplied by Bruker AXS, Karlsruhe, Germany). Hydrated Biodentine samples were manually ground with an agate mortar and pestle prior to XRD analysis. The PDFs used to identify the phases present in the anhydrous and hydrated Biodentine samples are listed in Table 2. The crystallinity of the samples and quantitative analysis of the anhydrous Biodentine sample by Rietveld refinement analysis [60] were obtained using TOPAS version 4.2 software (Bruker AXS, Karlsruhe, Germany). Crystallinity was calculated from the ratio of the area of the peaks of the crystalline phases to the total area of the signals arising from the crystalline and amorphous phases. A split pseudo-Voigt function was used to fit the broad signal arising from the amorphous phase. 

### 4.5. FTIR Spectroscopy

FTIR spectra of anhydrous and hydrated Biodentine samples were acquired using a Perkin Elmer Spectrum Two spectrometer ((Perkin Elmer, London, UK)) between 700 and 1900 cm^−1^ wavenumbers, with 10 scans at a resolution of 4 cm^−1^. Hydrated Biodentine samples were manually ground with an agate mortar and pestle prior to analysis. 

### 4.6. Isothermal Conduction Calorimetry

The rate of heat evolution during hydration of Biodentine was measured by isothermal conduction calorimetry using a Thermometric 2277 TAM (Thermometric AB, Stockholm, Sweden) calorimeter at 37 °C. In duplicate, ~0.06 g of accurately weighed Biodentine paste were placed in the calorimeter immediately after mixing. Power data were collected every second for 140 h. The rate of heat evolution was then calculated by dividing the power data by the total mass of Biodentine paste. 

### 4.7. ^29^Si MAS NMR Spectroscopy

MAS NMR spectra of anhydrous Biodentine powder and the hydrated samples were collected on a JEOL JNM-ECX 300 MHz spectrometer ((JEOL, Tokyo, Japan)). ^1^H-^29^Si cross polarization (CP) MAS NMR spectra were recorded with a contact time of 10^−3^ s, a pulse delay of 5 s, an acquisition time of 0.0256 s and a minimum of 50,000 scans. Single-pulse ^29^Si MAS NMR spectra were obtained with a pulse delay of 5 s, an acquisition time of 0.02048 s and a minimum of 90,000 scans. All spectra were collected with a spin rate of 6 kHz. ^29^Si chemical shifts were referenced to tetramethylsilane. The raw data were processed using Delta software (JEOL, Tokyo, Japan) to obtain spectra which were then analyzed and deconvoluted using Igor Pro software (WaveMetrics Inc., Portland, OR, USA). 

### 4.8. Deconvolution and Quantitative Analysis of ^29^Si MAS NMR Spectra

The ^29^Si MAS NMR spectrum of each hydrated Biodentine sample was analyzed according to the method reported by Love et al. [45]. The signal from the unreacted dicalcium and tricalcium silicate phases that obscures the resonances of the early Q^0^(H) signal was subtracted from the spectrum prior to deconvolution. This was achieved by adjusting the intensity of the ^29^Si MAS NMR spectrum of anhydrous Biodentine to match the intensity of the Q^0^ signals of the hydrated spectrum. The adjusted Biodentine background spectrum was then subtracted from the spectrum of the hydrated sample prior to deconvolution using iterative fitting of the Q^0^(H), Q^1^, Q^2B^ and Q^2 29^Si resonances to Voigt lineshapes. The relative abundance of the various Q^n^ species, mean silicate chain length (MCL) and degree of hydration were then calculated from the subtracted and deconvoluted spectra [46,47]. The formulae for the calculations of MCL and degree of hydration are given in Equations (3) and (4):

(3) $$\mathrm{MCL}=\frac{Q^{1}+Q^{2}+Q^{2B}}{\frac{1}{2}Q^{1}}$$

(4) $$\mathrm{Degree}\;\mathrm{of}\;\mathrm{hydration}=\frac{Q^{0}(H)+Q^{1}+Q^{2B}+Q^{2}}{Q^{0}+Q^{0}(H)+Q^{1}+Q^{2B}+Q^{2}}\times 100\%$$

where Q^n^ represents the intensity of the ^29^Si MAS NMR signal corresponding to the relevant silicate species. 

## 5. Conclusions

This study follows the early hydration chemistry of Biodentine endodontic cement by powder X-ray diffraction analysis (XRD), Fourier transform infrared spectroscopy (FTIR), ^29^Si magic angle spinning nuclear magnetic resonance spectroscopy (^29^Si MAS NMR) and isothermal conduction calorimetry. XRD analysis indicates that the anhydrous powder comprises 73.8 wt% triclinic tricalcium silicate, 4.45 wt% monoclinic β-dicalcium silicate, 16.6 wt% calcite and 5.15 wt% zirconium oxide. The formation of C-S-H gel is denoted by a progressive shift in the O-Si-O stretching vibrations from 905 to 995 cm^−1^ in the FTIR spectrum during 1 week. The extent of hydration was determined by ^29^Si MAS NMR to be 87.0%, 88.8% and 93.7% at 6 h, 1 day and 1 week, respectively, which is significantly higher than that of mineral trioxide aggregate (MTA). The mean silicate chain length (MCL) of the C-S-H gel was also estimated by this technique to be 3.7 at 6 h and 1 day, and to have increased to 4.1 after 1 week. This study demonstrates that ^29^Si MAS NMR spectroscopy is a valuable tool for the analysis of calcium silicate-based dental cements. It is particularly useful for the structural elucidation of the major C-S-H gel phase, which, owing to its poorly crystalline nature, is difficult to analyze by XRD and FTIR. 

## Figures and Tables

**Table 1:** Relative abundance of Q^n^ species, mean silicate chain length (MCL) and degree of hydration of Biodentine.

Sample	Q^0^ (%)	Q^0^(H) (%)	Q^1^ (%)	Q^2B^(%)	Q^2^ (%)	MCL	Hydration (%)
BDT-6	13.0	7.6	43.0	21.4	15.0	3.69	87.0
BDT-24	11.2	10.0	42.7	20.6	15.5	3.69	88.8
BDT-168	6.3	15.6	42.5	19.3	16.3	4.13	93.7

**Table 2:** PDF data used to identify crystalline phases in anhydrous and hydrated Biodentine.

Phase	Formula	PDF
Tricalcium silicate (triclinic)	Ca_3_SiO_5_	00-031-0301
Dicalcium silicate (monoclinic)	β-Ca_2_SiO_4_	00-033-0302
Zirconium dioxide (monoclinic)	ZrO_2_	00-013-0307
Calcium carbonate (calcite)	CaCO_3_	01-085-1108
Calcium hydroxide (hexagonal)	Ca(OH)_2_	01-073-6988
